# Self-Healing and Reprocessable Oleic Acid-Based Elastomer with Dynamic S-S Bonds as Solvent-Free Reusable Adhesive on Copper Surface

**DOI:** 10.3390/polym14224919

**Published:** 2022-11-14

**Authors:** Luca Pettazzoni, Francesca Leonelli, Andrea Giacomo Marrani, Luisa Maria Migneco, Fabrizio Vetica, Lorenzo Celio, Valerio Napoleone, Sara Alfano, Andrea Colecchia, Francesco Amato, Valerio Di Lisio, Andrea Martinelli

**Affiliations:** 1 Department of Chemistry, Sapienza University of Rome, 00185 Rome, Italy; 2 Donostia International Physics Center, Paseo Manuel de Lardizabal, 4, 20018 San Sebastian, Spain

**Keywords:** bio-based, self-healing, adhesive, disulfide, elastomer

## Abstract

In the last decade, the application of dynamic covalent chemistry in the field of polymeric materials has become the subject of an increasing number of studies, gaining applicative relevance. This is due to the fact that polymers containing dynamic functions possess a structure that affords reprocessability, recyclability and peculiar self-healing properties inconceivable for “classic” polymer networks. Consequently, the synthesis of a dynamic covalent chemistry-based polymer and its chemical, thermal, and mechanical characterizations are reported in the present research. In particular, oleic acid has been used as starting material to follow the founding principles of the circular economy system and, thanks to the aromatic disulfide component, which is the foundation of the material dynamic characteristics, the obtained polymer resulted as being reprocessable and self-healable. Moreover, the polymer can strongly interact with copper surfaces through the formation of stable Cu-S bonds. Then, the application of the polymer as a solvent-free reusable adhesive for copper was investigated by lap joint shear tests and comparisons with the properties of an analogous material, devoid of the disulfide bonds, were conducted.

## 1. Introduction

Recently, studies about innovative and “greener” plastic materials have gained more and more interest than ever, mainly because of the highly negative impacts that “classic” oil-based and barely recyclable plastics have on the environment [1,2,3]. Moreover, new research studies have been focused on the development of new kinds of polymers capable of overcoming the most notable limits of “classic” polymeric materials, such as short lifetime and difficult recycling processes [4,5]. A solution, aimed to go beyond these limitations, has been found in the inclusion into the polymeric structure of dynamic covalent bonds which confer a self-healing property, high reprocessability and recyclability, resulting in an extended material lifetime [6].

For this purpose, different types of dynamic covalent chemistry have been employed, including transesterification [7], transamidation [8], Diels-Alder/retro Diels-Alder system and aromatic disulfide metathesis [9,10,11]. The latter, in particular, has caught lot of attention, because of the wide availability of molecules containing aromatic S-S bonds suitable for polymer synthesis. Moreover, aromatic disulfide metathesis has proven to be effective in mild conditions and could occur with mild or without external stimuli [12,13].

A further step forward in the preparation of more sustainable polymeric materials can be pursued by using bio- or waste-derived materials for the monomer synthesis [14,15]. Therefore, in the present work, oleic acid has been used as the starting material for the synthesis of a dynamic polymer. This monosaturated omega-9 fatty acid is the major component of most of the vegetable oils and [16,17], more importantly from the sustainability point of view, it is one of the major constituents, in form of triglyceride, of waste cooking oils (WCO) and the major component of olive mill wastewaters [18,19,20]. The global annual production of WCO is estimated at around 16.5 million tons [21], representing one of the most critical hazardous agents for the aquatic environment [22]. Therefore, the use of WCO in the preparation of recyclable materials would be perfectly in line with the financing principles of the circular economy. In fact, oleic acid has already been used, both alone and in combination with other bio-based monomers, in the preparation of “classic” and dynamic polymers, resulting in the obtainment of materials with interesting mechanical properties, which vary depending on the polymer composition [8,23,24].

**Figure 1:**
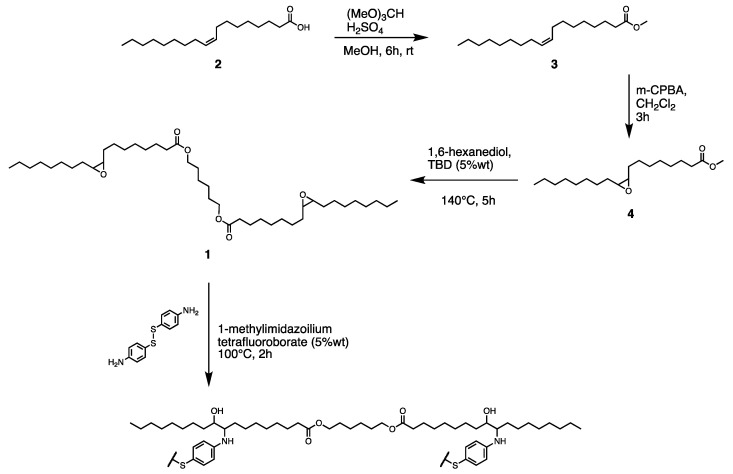
Synthetic route for the preparation of the monomer HMDO epox. **1** and polymerization reaction.

Hence, in this work oleic acid has been epoxidized and subsequently dimerized with 1,6-hexandiol, to form an aliphatic diester (HMDO epox. **1**, Figure 1). Then, HMDO epox. Has been polymerized via an epoxide ring opening reaction with 4-aminophenyl disulfide (4-ADS), a molecule able to donate a dynamic behavior to the polymeric structure due to the exchange process occurring between the disulfide bonds [25]. The thermal and mechanical properties of the obtained polymer (HMDO-ADS) were characterized by thermogravimetric analysis, differential scanning calorimetry and stress-strain experiments. Furthermore, its reprocessability and self-healing ability were investigated.

In addition to giving a dynamic character to the polymer chemical structure, the presence of disulfide bonds sparked interest in exploring a possible application of the polymer in a combined use with metals. In fact, it is well known that organo-thiols and organo-disulfide can be chemisorbed on gold, silver, and copper through the formation of metal-S bonds. Because of the high stability of the S−Cu, self-assembled monolayers formed by aliphatic and aromatic thiols or disulfides on copper have been shown to be a facile and effective method for the protection of metal surfaces from corrosion and as adhesion promoters [26,27,28]. Moreover, amine- and hydroxyl-functionalized thiols and disulfide have been used as coupling agents for the adhesion of epoxy resins on copper through covalent bonding, resulting in an increased adhesion, even at elevated temperatures [29,30,31]. Accordingly, the chemisorption of HMDO-ADS on a copper surface through the formation of an Cu-S bond was investigated by water contact angle and FTIR analyses. The specificity of the polymer interaction with the metal was ascertained by comparing the experimental results with those obtained from an analogous polymer prepared from HMDO epox. and 2,2’-ethylenedianiline, i.e., devoid of the disulfide bonds. Then, the application of the polymer as a solvent-free adhesive for copper was investigated by lap joint shear tests at room temperature. The adhesion was observed to be promoted by heating the joint at 150 °C and by applying a mild pressure. Additionally, once cohesion failure occurred, the repeatedly reformed joint showed a small reduction in shear stress.

## 2. Materials and Methods

### 2.1. Materials

Oleic acid (90%), 4-aminophenyl disulfide (98%), 2,2′-ethylendianiline (97%), trimethyl orthoformate (99%), 1,6-hexanediol (97%), 1-methylimidazole (99%), *meta*-chloroperoxybenzoic acid (77%), 1,5,7-triazabicyclo[4.4.0]dec-5-ene (98%), dichloromethane (≥99%), ethyl acetate (≥99%), diethyl ether (≥99%), hexane (≥99%), methanol (≥99%), ethanol (96%), tetrahydrofuran (99%), tetrafluoroboric acid (57% in Et_2_O), sulfuric acid (99%), NaCl (≥99%), NaHCO_3_ (≥99%), Na_2_SO_4_ (≥99%) and Na_2_SO_3_ (≥99%) were purchased from Sigma-Aldrich Co., St. Louis, MO, USA, and all of them were used without further purification. All solvents used were of technical and analytical grade. Flash silica (High-purity grade, pore size 60 Å, 230–400 mesh particle size, 40–63 μm particle size, Sigma-Aldrich Co.) was used for purification. Thin-layer chromatography (TLC) was carried out by using silica gel on TLC plates (5 cm × 20 cm, silica gel matrix, fluorescent indicator, Sigma-Aldrich Co.) 

### 2.2. Preparations

#### 2.2.1. Methyl Oleate (**3**) Preparation [32]

Oleic acid (**2**) (20 g, 0.071 mol), methanol (20 mL), trimethyl orthoformate (2.8 mL) and sulfuric acid (0.6 mL) were mixed in a round-bottomed flask and heated until reflux, under stirring, for 6 h. The reaction was monitored by thin-layer chromatography (TLC) (2:8 = diethyl ether:hexane). Once ended, the crude product was solubilized with Et_2_O and, sequentially, washed with water, a NaHCO_3_ saturated solution, and brine. Then, the organic phase was dried over anhydrous Na_2_SO_4_ and filtered. Eventually, the solvent was removed by rotary distillation under vacuum. Methyl oleate (MO, **3**) was then obtained quantitatively (98% of conversion) as a light-yellow oily liquid, confirming the compound structure through NMR analysis.

^1^H-NMR (400 MHz, CDCl3, δ in ppm): δ = 5.39–5.30 (m, -HC=CH-), 2.32–2.28 (t, -CH_2_-COO-), 2.05–1.98 (m, -CH_2_-CH=), 1.65–1.58 (m, -CH_2_-CH_2_-COO-), 1.30–1.26 (m, -CH_2_-), 0.89–0.86 (t, -CH_3_). ^13^C NMR (100 MHz, CDCl3, δ in ppm): δ = 174.0 (-COO-), 130.2 (-CH=CH-), 64.3 (CH_3_-OCO-), 34.5–22.8 (-CH_2_-), 14.2 (-CH_3_). 

#### 2.2.2. Methyl Oleate Epoxidized (**4**) Preparation

Methyl oleate (**3**) (10 g; 0.033 mmol) was weighed in a round-bottomed flask and 60 mL of dichloromethane were added. The mixture was stirred using a magnetic stir bar and cooled in a 0 °C ice/water bath. M-chloroperbenzoic acid (8.72 g; 0.050 mol) was then slowly added over 5 min, the ice bath was removed, and the reaction was allowed to stir for 3 h. Then, the crude product was transferred in a separating funnel with dichloromethane and washed, sequentially, with a Na_2_SO_3_ saturated solution (to quench unreacted peroxides), a NaHCO_3_ saturated solution, and brine until neutralization. The organic phase was dried over anhydrous Na_2_SO_4_, filtered, and the solvent removed by rotary distillation under vacuum. The crude product was isolated by filtering through flash silica, using an 8%_v.v._ ethyl acetate/hexane mixture as eluent. The solvent was removed by rotary distillation under vacuum. Methyl oleate epoxidized (MOE, **4**) was then quantitatively obtained (97% of conversion) as a colorless liquid, confirming the compound structure through NMR analysis.

^1^H NMR (400 MHz, CDCl3, δ in ppm): δ = 3.66 (s, -OMe), 2.94–2.82 (m, -CH-O-CH-), 2.30 (t,-CH2-COO-), 1.66–1.57 (m, -CH2-CH2-COO-), 1.51–1.43 (m, -CH2-CH-O-), 1.36–1.23 (m, -CH2-), 0.87 (t, -CH3). ^13^C NMR (100 MHz, CDCl3, δ in ppm): δ = 174.0 (-COO-), 64.2 (CH_3_-OCO-), 57.5 (-CH-O-CH-), 34.5–22.8 (-CH_2_-), 14.2 (-CH_3_).

#### 2.2.3. 1,6-Hexanediyl Dioleate Epoxidized (**1**) Synthesis

Methyl oleate epoxidized (**4**) (10 g, 0.032 mmol), 1,6-hexanediol (1.92 g, 0.016 mmol) and 1,5,7-triazabicyclo[4.4.0]dec-5-ene (TBD) (0.5 g, 5%_w.w._ of MOE) were mixed in a round-bottomed flask and heated up to 140 °C, under stirring. The reaction was monitored by thin-layer chromatography (TLC) (1:9 = ethyl acetate:hexane). After 5 h, the crude product was solubilized with dichloromethane and washed with brine. Then, the organic phase was dried over anhydrous Na_2_SO_4_, filtered and the solvent was removed by rotary distillation under vacuum. The crude product was isolated by filtration on flash silica with a 10%_v.v._ ethyl acetate/hexane eluent solution. Then, 1,6-Hexanediyl dioleate epoxidized ester (HMDO epox., **1**) was obtained in an 89% yield, according to NMR analysis. It appears as a white solid.

^1^H-NMR (400 MHz, CDCl3, δ in p pm): δ = 4.07–4.04 (t,-CH_2_-OCO-), 2.92–2.87 (m,-CH-O-CH-), 2.31–2.27 (t, -CH_2_-COO-), 1.65–1.60 (q,-CH_2_-CH_2_-COO-), 1.43–1.36 (m, -CH_2_-CH_2_-OCO-), 1.32–1.27 (d, -CH_2_-), 0.90–0.86 (t, -CH_3_). ^13^C-NMR (100 MHz, CDCl3, δ in ppm): δ = 174.0 (-COO-), 64.2 (-CH_2_-OCO-), 57.4 (-CH-O-CH-), 34.5–22.8 (-CH_2_-), 14.1 (-CH_3_).

IR (ATR): $\underline{\nu }$ = 1724 (C=O stretch), 1211 (epoxide symmetric stretch), 847 (epoxide asymmetric stretch) cm^−1^.

#### 2.2.4. 1-Methylimidazolium Tetrafluoroborate Preparation [33]

Tetrafluoroboric acid (57% in Et_2_O, 0.83 mL) was slowly added to 1-Methylimidazole (0.5 g, 6.1 mmol) at 0 °C, under mild stirring, in about 30 min with a dropping funnel in a two-neck round-bottomed flask. The reaction mixture was left at room temperature for 2 h under strong stirring. The obtained product was dried in vacuum and used without further purification.

#### 2.2.5. Polymerization Procedure

The polymer (HMDO-ADS) was prepared by mixing in a vial 300 mg of HMDO epox. (0.44 mmol), 109 mg of 4-aminophenyl disulfide (0.44 mmol) and 1-Methylimidazolium tetrafluoroborate (15 mg, 5 wt%) at 100 °C under argon flux, to prevent oxidation phenomena. After 5 min, the viscous homogeneous mixture was poured in a Teflon mold and let to react for 2 h at 100 °C in Ar atmosphere. Then, after cooling at RT, the polymer was removed.

The analogous polymer without disulfide bond was obtained by polymerizing in the same conditions HMDO-epox. (300 mg, 0.44 mmol), 2,2’-Ethylenedianiline (DAE, 94 mg, 0.44 mmol) and 1-Methylimidazolium tetrafluoroborate (15 mg, 5 wt%) to give HMDO-DAE.

### 2.3. Methods

#### 2.3.1. Solubility in Organic Solvent

The polymer solubility was evaluated by measuring the gel fraction (*G_F_*) and the swelling ratio (*S_R_*) of the polymers. Samples were weighed (*w*_0_) and then immersed in the solvent of choice (ethyl acetate, methanol and tetrahydrofuran). The insoluble parts were then weighed (*w*_1_), dried in an oven at 40 °C for 24 h and weighed again (*w*_2_). *G_F_* and *S_R_* and were calculated through the following equations:

(1) $$G_{F}=\frac{w_{2}}{w_{0}}\times 100$$

(2) $$S_{R}=\frac{w_{1}-w_{2}}{w_{2}}$$

#### 2.3.2. Thermal Analysis

The sample thermal behavior was investigated by differential scanning calorimetry (DSC) and thermogravimetric analysis (TGA). DSC thermograms were obtained by heating about 4–6 mg of sample from −30 °C to 200 °C. Then, the sample was cooled to −30 °C and reheated at 200 °C. All the scans were carried out at 10 °C min^−1^ under N_2_ flow (30 mL min^−1^). A DSC profile was also acquired in isothermal mode, by annealing HDMO-ADS sample for 1 h at 150 °C under an air flow of 30 mL min^−1^. At the end of the heat treatment, the sample was analyzed by ATR-FTIR. Thermogravimetric analysis (TGA) was performed using a Mettler TG50-MT5 with a heating rate of 10 °C min^−1^ from 25 °C to 500 °C under N_2_ flow (30 mL min^−1^).

#### 2.3.3. Material Reprocessing

Reprocessability tests were carried out by cutting the polymers into small pieces, which were then inserted into a hot press heated to 150 °C for 30 min, under a pressure of 98 MPa.

#### 2.3.4. Tensile Test

Stress-strain tests on pristine and self-healed samples were carried out by a universal testing machine Instron 4502 at room temperature, using a 2 kN load cell at a crosshead speed of 5 mm min^−1^. The dumbbell specimens were prepared by polymerizing the sample directly in a Teflon mold, characterized by the shape of C-type die of ASTM D 412 method with dimensions reduced by about a 0.5 factor to accommodate the mold in the reaction vessel. The dimensions of the resulting polymer specimens were: overall length 51 mm, narrow Section 3 mm wide and 18 mm long. The thickness of the specimens was about 2 mm. After the sample rupture occurring in the stress-strain experiments, the break surfaces were brought into contact and the specimen heated for 30 min at 100 °C in the same mold. Then, the healed specimens were subjected to a second stress-strain experiment in the same condition used for the pristine ones.

#### 2.3.5. Adhesion Test and Contact Angle

In order to evaluate the adhesion of polymer samples on copper surface, adhesive lap joint shear tests were carried out. The edge of copper of strips (10 × 40 mm^2^) was first etched according to the procedure described in reference [19]. Between the etched zone (10 × 10 mm^2^) about 200 mg of HMDO-ADS and HMDO-DAE, used as control, were compressed at 360 KPa for 15 min at 25, 60, 100, 150 and 200 °C. The test specimens were then cooled at RT and pulled in tension at 5 mm min^−1^ in long axis direction by an Instron 4502 universal testing machine. After the joint failure, the maximum shear strength was calculated by the ratio of the maximum recorded load and the adhesion area. Then, the sample surface was visually observed. The clean detachment of the sample from the Cu substrate was indicative of adhesive failure, whereas the presence of polymer on the metal surface was indicative of cohesive failure. Polymer self-healing behavior was evaluated by re-forming 4 times the lap joint at 150 °C of the already fractured test specimens.

The involvement of sulfur-copper bond in specific adhesion of HMDO-ADS on the metal surface was investigated by dynamic contact angle analysis, FTIR and Raman spectroscopy. The etched copper surface was soaked in ethyl acetate (AcOEt) containing the HMDO-ADS and HMDO-DAE soluble fraction. Then, in one procedure, the solution on the copper strips was dried to have a homogeneous film on the metal surface (HDMO-ADS film and HDMO-DAE film). In the second procedure, after the immersion the samples were rinsed three times with AcOEt and sonicated for 15 min in AcOEt (Cu-HMDO-ADS and Cu-HMDO-DAE samples). This procedure was carried out also by soaking the etched copper strip in 4-ADS solution (Cu-ADS sample). The dried samples were then analyzed by dynamic contact angle measurements by using water (milli-Q water, γ_w_ = 73 ± 1 mN m^−1^) as wetting medium and a DCA-312 dynamic contact angle analyzer (CAHN Instruments, Inc., Cerritos, CA, USA) at a stage speed of 40 μm s^−1^. The advancing contact angle results are reported as mean value ± standard deviation evaluated on three repetitions.

#### 2.3.6. FTIR Spectroscopy

Attenuated total reflection FTIR (ATR-FTIR) spectra were obtained using a Nicolet 6700 Fourier transform infrared spectrophotometer (Thermo) equipped with a Golden Gate ATR device (Specac). The spectra were recorded from 4000 to 600 cm^−1^, at 4 cm^−1^ resolution and 200 scans.

The FTIR characterization of Cu-HMDO-ADS and HMDO-ADS film samples were carried out in external reflection mode by using an incident angle of 80°, an etched copper strip for the background and 1000 scans per spectrum at 4 cm^−1^ resolution.

#### 2.3.7. NMR Spectroscopy

The ^1^H-NMR and ^13^C-NMR spectra were recorded by a Bruker AVANCE™-400 spectrometer (400.13 MHz, 100.61 MHz). All NMR spectra were acquired in CDCl_3_, and the chemical shifts were expressed in ppm (δ) relative to the residual solvent peak (7.26 ppm for ^1^H-NMR and 77.2 ppm for ^13^C-NMR).

#### 2.3.8. Thin-Layer Chromatography

Thin-layer chromatography (TLC) was performed to follow the progress of the reaction, using YLC plates coated with a stationary phase of silica and endowed with a fluorescent indicator. The spots were revealed with the use of both UV light (254 nm) and a solution of phosphomolybdic acid in ethanol (10% _w/v_).

## 3. Results and Discussion

### 3.1. HMDO-ADS Synthesis and Swelling Tests

**Figure 2:**
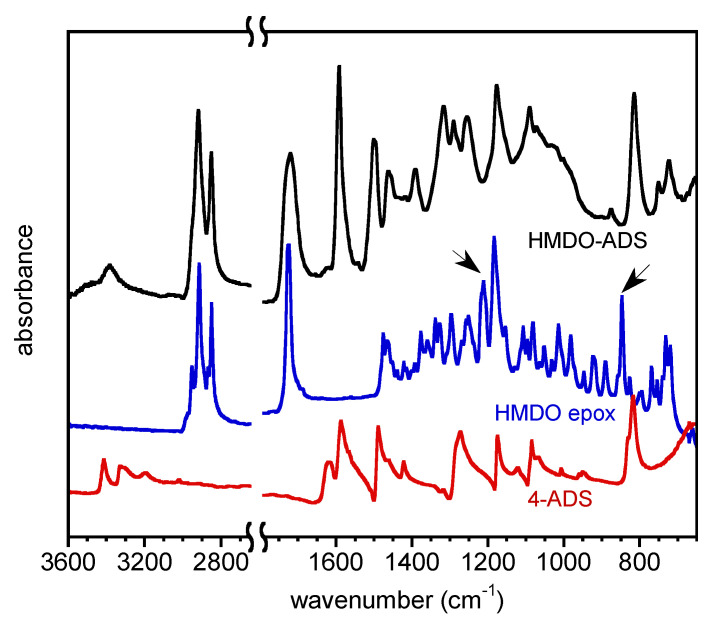
FTIR spectra of 4-ADS, HMDO epox. and HMDO-ADS.

The use of a catalytic amount of 1-Methylimidazolium tetrafluoroborate is required for the epoxide ring opening reaction by the aromatic nitrogen of the 4-aminophenyl disulfide, that otherwise would not take place. The extent of polymerization was followed by ATR-FTIR analysis, monitoring the epoxide signal disappearance. As shown in Figure 2, where the spectra of the two monomers (4-ADS and HMDO epox.) and the polymer (HMDO-ADS) are reported, the signals relative to the epoxide asymmetric stretching at 847 cm^−1^ and symmetric stretching at 1211 cm^−1^ (marked with arrows) of HMDO monomer completely disappeared after 2 h, at the end of the polymerization. Moreover, in the 3300–3500 cm^−1^ range of the HMDO-ADS spectra, the appearance of the bands relative to the O-H and N-H stretching arising from the epoxide ring opening reaction can be observed.

The HMDO-ADS and HMDO-DAE underwent swelling tests to verify their solvent resistance. The values of gel fraction (*G_f_*) obtained for the material, reported in Table 1, indicate that the samples are partially soluble in the selected organic liquids, that are all good solvents for HMDO epox. and the 4-aminophenil disulfide.

However, the low solubility in the chosen solvents let us infer that the polymer is partially cross-linked. This occurs because some of the nucleophilic nitrogen of 4-ADS react with two epoxides rather than one, creating cross-links and leaving some primary amine unreacted. To confirm this hypothesis, two different experiments were performed.

**Figure 3:**
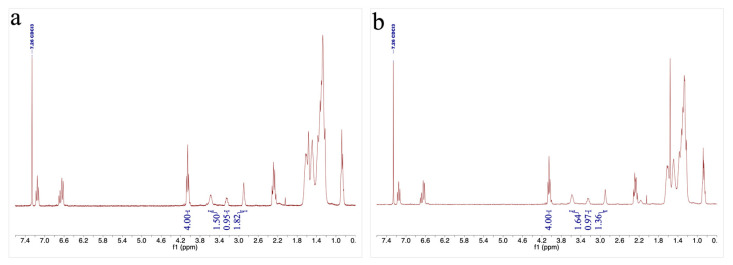
^1^H-NMR spectra of the reaction between HMDO epox. and aniline after 2 (**a**) and 4 (**b**) hours.

In the first one, HMDO epox. was reacted with one equivalent of aniline (epoxide/aniline = 2/1), used as aromatic amine model, at 100 °C for 4 h. The reaction was monitored through ^1^H-NMR spectroscopy by acquiring a spectrum of the crude every two hours. By checking the values of the integral of the epoxide signal at 2.90 ppm in the ^1^H-NMR spectra and taking the value of the integral of the -CH_2_-OCO as a reference, we observed that after two hours the 55% of the epoxide function was consumed (Figure 3a), while after 4 h this percentage increased until 66% (Figure 3b). At the same time, it was also possible to observe a growth of the integrals of the -CH- signals deriving from the epoxide ring opening (3.58 and 3.24 ppm). Through this evidence it was possible to confirm that a single secondary aromatic amine generated by the reaction of an aniline with an epoxide can react further with an additional epoxide function, thus giving a tertiary aromatic amine.

In the second experiment, in order to assess if this reaction had taken place during the polymer synthesis, the presence of primary aromatic amines in the polymer was evaluated by a qualitative test carried out on the HMDO-ADS soluble fraction in ethyl acetate. Upon addition of nitrous acid, N_2_ gas evolution was observed. Marking the decomposition of the aromatic diazonium salts, and thus confirming the presence of primary, unreacted, aromatic amines.

### 3.2. Self-Healing and Reprocessability

The ability of HMDO-ADS to self-heal or to be reprocessed is one of the materials most important features, which is given by the use of 4-aminophenyl disulfide in its preparation.

**Figure 4:**
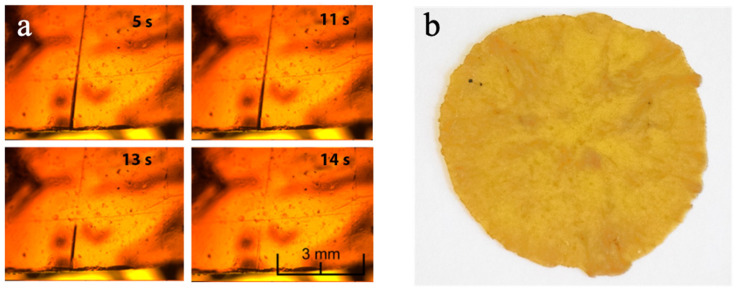
(**a**) Image sequence of self-healing process of HMDO-ADS recorded by optical microscope at 40× magnification. (**b**) Image of reprocessed HMDO-ADS film.

This means that a cut in the material can be easily and readily repaired just by putting in contact the two surfaces at room temperature or in neutral-to-mild conditions. This is made possible by the dynamic nature of the aromatic disulfide bridges, which can undergo exchange reactions at room temperature through a radical mediated [2+1] mechanism [34]. This property was directly investigated by monitoring the evolution of a small cut on a thin film of HMDO-ADS by optical microscope (40× magnification) at room temperature. The images acquired as a function of time, reported in Figure 4a, show the progressive disappearance of the fracture line of HMDO-ADS sample, evidencing the rapid self-healing process that ends within about 14 s. For sake of comparison, the same experiment was carried out on and HMDO-DAE which, in contrast to HMDO-ADS, did not show any healing of the cut.

Furthermore, the reprocessability of HDMO-ADS was investigated by dicing the polymer into small pieces which were then pressed at 120 °C for 30 min under a pressure of 98 MPa. The obtained film, reported in Figure 4b, was homogeneous as the pristine materials.

**Figure 5:**
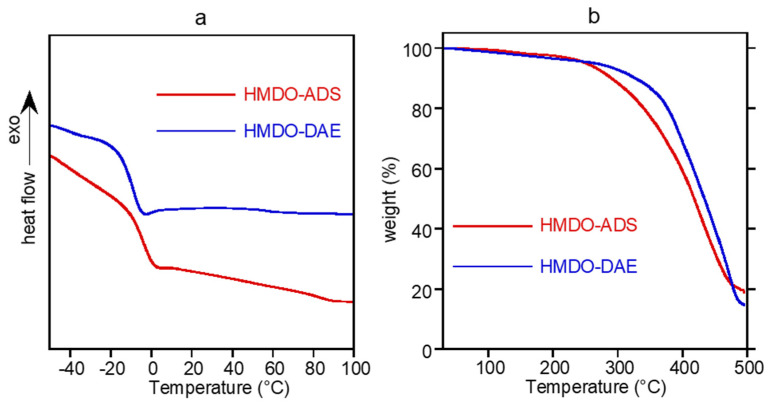
DSC curves (**a**) and thermograms (**b**) of HMDO-ADS and HMDO-DAE.

The thermal behavior of the sample was investigated by differential scanning calorimetry, heating two times the sample between −50 °C and 200 °C. The DSC curves of the first scan are reported in Figure 5a. In the explored temperature range, the HMDO-DAE shows a glass transition at −11 °C, few degrees below that of HMDO-ADS (T_g_ = −6 °C). The higher T_g_ of polymer containing disulfide bonds is due to its higher crosslinking density, highlighted by its lower swelling ratio with respect to that of the HMDO-DAE sample. No different thermal behavior was observed in the second heating. Hence, at room temperature, all the samples are in rubbery state. Thermogravimetric analysis was used to evaluate the thermal stability of the polymer, mainly in view of the adhesion experiments, when the sample are subject to repeated heating. In Figure 5b, the thermograms of HMDO-ADS and HMDO-DAE sample are reported. Both the polymers showed good heat stability, being about 5 wt% the weight loss up to 250 °C. The lower degradation temperature of HMDO-ADS compared to that of HMDO-DAE is due to the presence of more labile S-S bonds. In order to evaluate an oxidation process possibly occurring at the expense of the disulfide group, the HMDO-ADS sample was annealed at 150 °C for 1 h under an air flux of 30 mL/min. During the process, which was followed by DSC, no exothermic events were recorded. The FTIR analysis of the annealed sample, which did not reveal any spectrum change respect to the pristine one, excluded the possible oxidation process at the expense of disulfide group [35]. Therefore, the results showed that the polymer sample can be safely heated at high temperature without any modification of its chemical structure and physical properties.

### 3.3. Contact Angle Results

In order to verify the possible specific interaction between the S-S bonds of HMDO-ADS with copper surface, different samples, obtained as described in the Section 2.3.5., were prepared and analyzed by measuring the contact angle in water. In Table 2, the mean values (*n* = 3 ± SD) are reported.

The results of the contact angle analysis show that both the HDMO-DEA and HMDO-ADS are lightly hydrophobic, showing a contact angle higher that 90°. Moreover, the contact angle of bare copper changes as a result of the 4-ADS absorption through the sulfide-Cu bond which led to a slight wettability decrease. More indicative are the differences between Cu-HMDO-ADS and Cu-HMDO-DAE samples. The high contact angle of Cu-HMDO-ADS evidences the formation of specific and stable interaction between the metal substrate and the polymer via Cu-S bond formation. It is interesting to highlight that the contact angle of Cu-HMDO-ADS sample is significantly higher than that of the film obtained from the same polymer. This difference could be due to the structure of the chemisorbed polymer chains that, interacting with the copper substrate through the aromatic sulfide, are forced to expose the hydrophobic alkyl moiety of oleic acid. Differently, the influence of the copper–polymer interface vanishes on the thick film surface, which assumes a lower contact angle. The contact angle of Cu-HMDO-DAE sample, equal to that of bare copper, evidenced that the adsorbed polymer was completely removed by rinsing, lacking functional groups able to react with the metal surface.

### 3.4. FTIR Spectroscopy

**Figure 6:**
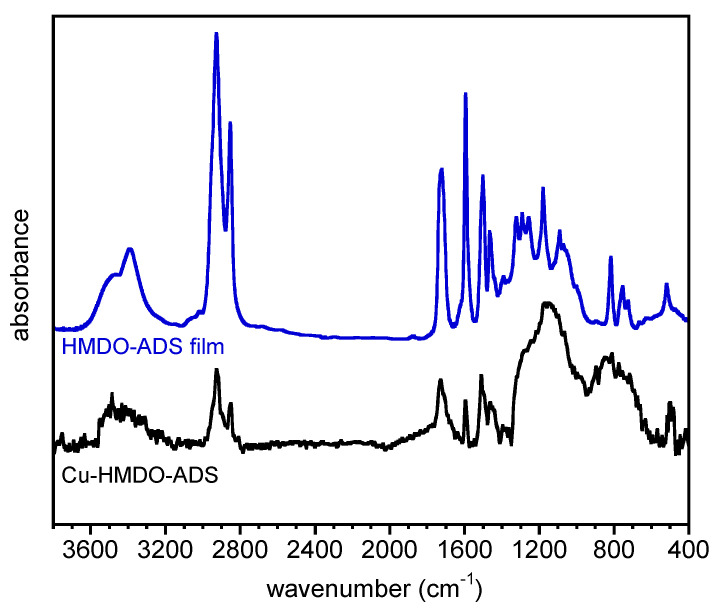
FTIR spectra of HMDO-ADS film and Cu-HMDO-ADS. The absorbance of Cu-HMDO-ADS spectrum was multiplied by a factor 10.

The interaction of HMDO-ADS with copper surface was further investigated by FTIR spectroscopy. In Figure 6, the baseline corrected spectra of HMDO-ADS film and Cu-HMDO-ADS samples, obtained in external reflection mode, are reported. For sake of comparison, the absorbance of the Cu-HMDO-ADS spectrum is multiplied by a factor 10.

The thin layer of polymer obtained after rinsing the copper substrate resulted in a very weak and noisy signal from which, however, the presence of HMDO-ADS is clearly visible, even if some bands between 1200 and 450 cm^−1^ were poorly resolved [36]. The expected absorption of the S-Cu stretching, located at about 200–280 cm^−1^, is out of the explored wavelength region. However, as for other metal-S bonds, this vibration is quite elusive and often not observed [26,37].

### 3.5. Mechanical Characterization

**Figure 7:**
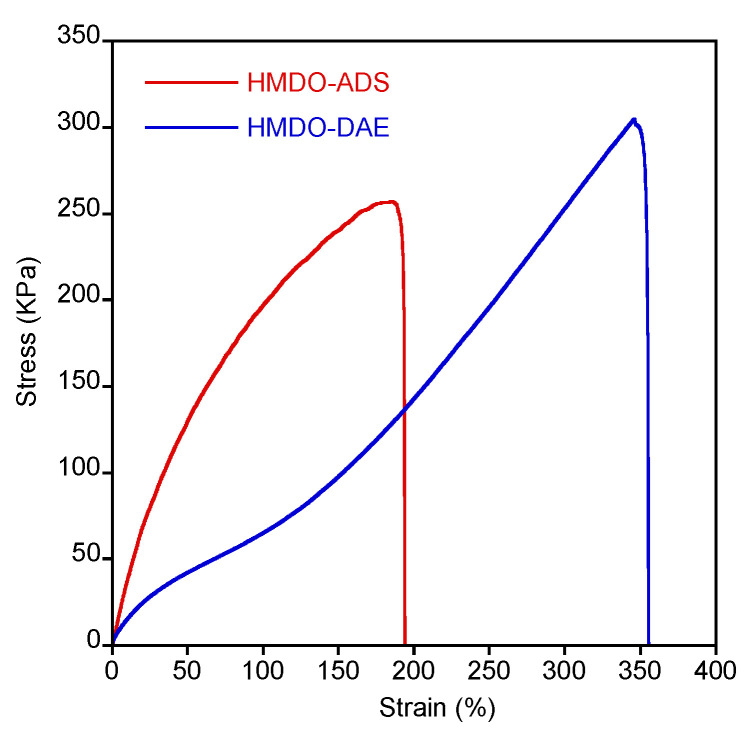
Representative stress-strain curves of HMDO-ADS and HDMO-DAE samples.

As aforementioned, a specific interaction of HMDO-ADS with copper surface through the formation of S-Cu bond has been found. To exploit this behavior and employ the polymer as a potential reusable adhesive, the material must have the appropriate resistance to mechanical stresses. Then, the mechanical properties of HMDO-ADS and HMDO-DAE samples were analyzed by stress-strain experiments whose selected results are reported in Figure 7.

**Figure 8:**
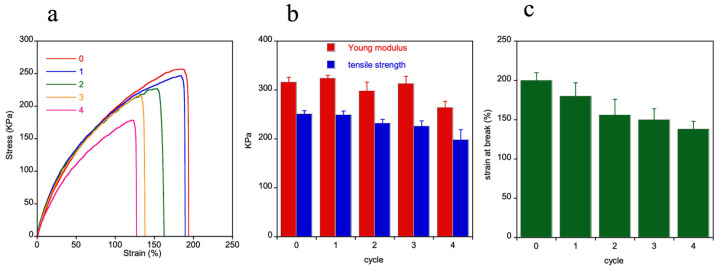
HMDO-ADS mechanical properties. Representative stress-strain curves of HMDO-ADS sample obtained in subsequent four healing cycles (**a**). Young modulus, tensile strength (**b**) and strain at break (**c**) as a function of cycle number.

The two polymers clearly show different mechanical behavior. In fact, HMDO-ADS resulted more rigid and broke at lower deformation than HMDO-DAE, which shows a typical elastomeric behavior with high tensile strength and elongation at break. After the rupture of the dumbbell shaped specimens, the broken surfaces were brought together into the Teflon mold and heated at 150 °C for 30 min. No surface welding was obtained for the HMDO-DAE sample, while the healing process occurred for the HMDO-ADS. Then, the broken specimens were healed and tested for four times consecutively, and the results are reported in Figure 8.

All the samples broke without showing irreversible shear phenomena and rapidly regained the original dimensions after the rupture. From the stress-strain experiments, the Young modulus, calculated as the slope of the curves in the 0.05–0.2 strain range, the strain at break and the tensile strength were evaluated for each cycle (Figure 8b,c). The mechanical properties of the samples show only a slight drop after the healing process, mainly in the last cycle, with the strain at break displaying the largest decrease. In fact, it was observed that the sample failure occurred always in the same zone, presumably because of an imperfect surface overlapping which brought about the presence of joint defects, as voids or surface crevices, that trigger the crack propagation.

**Figure 9:**
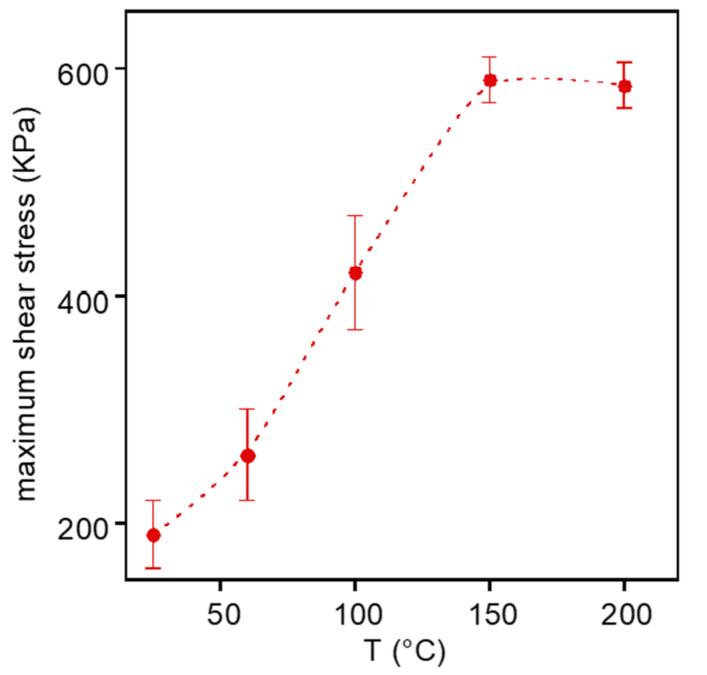
Maximum shear strength of copper-HMDO-ADS joint as a function of the specimen preparation temperature.

Then, lap joint shear tests were carried out to investigate the adhesive performances of HMDO-ADS. Preliminary tests were carried out to find the optimal temperature to prepare the specimens. In Figure 9, the maximum shear strength is reported as a function of the temperature used to press at 360 KPa HMDO-ADS between the two etched copper strips.

The joint obtained at 25 °C failed adhesively; at 60 °C and 100 °C the failure was mixed, adhesive and cohesive; while at 150 and 200 °C it was only cohesive. Then, since the sample was found to be thermally stable and showed the highest maximum shear strength at 150 °C and 200 °C, all the junctions were prepared at 150 °C. The polymer heating favors its softening and, hence, the intimate contact with the substrate at low pressure as well as the mobility necessary for the surface reaction between the disulfide bond and copper. It is important to underline that, unlike HMDO-DEA, HMDO-ADS is characterized by a relatively high cross-linking density and that it can irreversibly flow between the metal surface thanks to the dynamic S-S bond which, at high temperature, undergoes cleavage and reformation at room temperature. Differently, it can be presumed that the conformability of HMDO-DEA is mainly due to the flow of macromolecules not involved in the network.

The influence of preferential adhesion of HMDO-ADS polymer on metal surface was then investigated by comparing the results of adhesive lap joint test to those obtained by using HMDO-DAE sample. In Table 3, the maximum shear strength of the samples is reported.

It shows that the viscoelastic behavior and wetting property of both the polymers toward the substrate favor their adhesion on the metal, typically through van der Waals non-specific interactions. However, the resistance of the joint prepared with HMDO-ADS is significantly higher than that with HMDO-DAE. Despite this, its failure was always cohesive, as proof of the strong adhesion of the polymer to copper surface mediated by Cu-S bonds. Otherwise, the joint prepared with HMDO-DAE failed due to the complete detachment of the polymer from the metal.

The specific interaction of the sulfide bond with copper was also investigated by performing the adhesive lap joint shear tests on aluminum strips in the same condition used for the copper substrate. The results, reported in Table 3, evidenced that the maximum shear strength was always significantly lower on this metal and that the failure is always adhesive.

**Figure 10:**
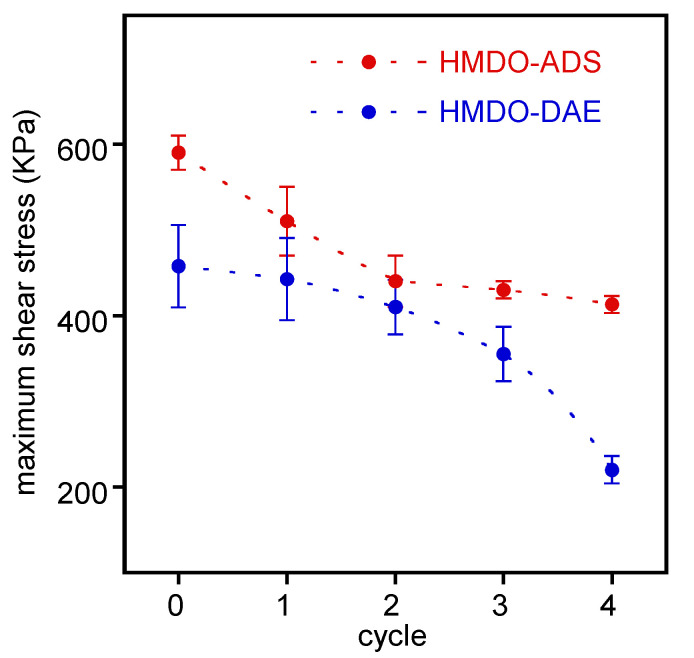
Maximum shear strength of copper strips joined at 150 °C five times with HMDO-ADS and HMDO-DAE polymers.

All collected properties of HMDO-ADS, including self-healing, stable adhesion on copper and cohesive failure, can be exploited by using the polymer as adhesive. In order to test its reusability, four successive cycles of lap joint rupture and reformation were analyzed. In each cycle the failure zone was matched, pressed and heated in the same conditions used in the first lap joint preparation. In Figure 10, the maximum shear strength values of copper specimens prepared with HMDO-DAE and HMDO-ADS are reported as a function of cycle number.

The strength trend of the joint prepared with HMDO-ADS shows a slight decrease in each subsequent experiment. Moreover, the failure mode is always cohesive. On the other hand, the maximum shear strength of the lap joint obtained with HMDO-DAE undergoes a progressive and considerable decrease, failing constantly at the interface between polymer and metal.

The HMDO-ADS polymer has shown properties adequate for its use as reusable adhesive, particularly on copper substrates. In fact, thanks to the formation of S-Cu bonds, it showed a strong adhesion on this metal, higher than its cohesive resistance. Moreover, at room temperature, HMDO-ADS is in the rubbery viscoelastic state and absorbs mechanical stresses occurring at the polymer-metal interface but easily conforms to the metal surface at high temperature. Furthermore, it has also been observed that HMDO-ADS can be reprocessed, healed and reused multiple times thanks to the exploitation of the dynamic character of the aromatic disulfide bond. These features are advantageous from an applicative point of view in that, differently from conventional fluid glues, HMDO-ADS can be obtained as thin film which can be easily and directly placed on the surfaces to be joined without the necessity of solvents or of metal pretreatments, as the addition of adhesion promoters [38].

The comparison of the adhesion properties of HDMO-ADS with other pressure adhesives is difficult in that very wide material typology, application, substrate and mechanical properties are described in literature. However, the obtained shear stress is in line with other pressure sensitive adhesive endowed with dynamic bonds [39,40,41].

Furthermore, it was not actually possible to determine the maximum bond strength between the polymer and the copper surface due to the occurrence of cohesive failure of the lap joint. However, the analysis of the chemical structure of HMDO-ADS allows us to foresee the possibility to obtain materials with different mechanical properties. The synthetic route allows for the preparation of a polymer with a higher cohesion by increasing the crosslinking density through the use of higher amount of epoxidized monomer which can further react with secondary amine, as well as by adding a reinforcing filler or using other bio- or waste-derived monomers that possess more than two epoxide functions [42,43,44]. Moreover, the viscoelastic and thermal properties of HMDO-ADS could be modulated by employing diol of different length and flexibility, possibly able to modulate the glass transition temperature. More research in this context is in progress.

## 4. Conclusions

In this work, the preparation, the characterization and the possible application of an innovative, oleic acid-based, green polymer (HMDO-ADS) are reported. The monomer (HMDO epox.) for the polymer synthesis was obtained from oleic acid (obtainable from food waste), thus reducing the environmental impact of the material, while the presence of the aromatic S-S bonds in the polymer structure allows for the achievement of a rapid self-healing ability of a fresh cut at room temperature and reprocessability at a higher temperature. Moreover, the application of HMDO-ADS as an adhesive for copper was investigated. At first, the stable interaction between HMDO-ADS and copper via a Cu-S bond was found by contact angle analysis and FTIR spectroscopy. Thereafter, lap joint shear tests highlighted a strong adhesion of HMDO-ADS on copper substrate, higher that that recorded by using HMDO-DAE, an analog polymer without dynamic S-S bonds. Moreover, it was evidenced that the lap joint prepared with HMDO-ADS could undergo repeated rupture and reformation cycles with a small shear stress drop. In summary, in HMDO-ADS, the healing ability and the metal adhesion properties of the S-S bond were combined in a unique polymeric material, resulting in the preparation of an innovative, reusable and bio-based adhesive.

## Figures and Tables

**Table 1:** *S_R_*, *G_F_* and obtained in the chosen solvents for HMDO-ADS and HMDO-DAE.

Polymer	Liquid	*S_R_*	*G_F_* (%)
HMDO-ADS	THF	9.95	91
	MeOH	0.28	98
	AcOEt	2.73	80
HMDO-DAE	AcOEt	10.8	75

**Table 2:** Advancing contact angle in water of samples on copper strips.

Sample	Advancing Contact Angle (°)
Bare copper	78 ± 5
HMDO-ADS film	93 ± 2
HMDO-DAE film	97 ± 5
Cu-ADS	92 ± 3
Cu-HMDO-ADS	109 ± 2
Cu-HMDO-DAE	77 ± 5

**Table 3:** Maximum shear strength of HMDO-ADS and HMDO-DAE samples on copper and aluminum substrates.

Sample	Maximum Shear Strength (KPa)	Failure	Maximum Shear Strength (KPa)	Failure
substrate	Cu		Al	
HMDO-DAE	460 ± 50	adhesive	300 ± 50	adhesive
HMDO-ADS	590 ± 20	cohesive	350 ± 10	adhesive

## Data Availability

Data supporting the finding of this study are available from the corresponding authors upon reasonable request.
